# Delayed ejaculation and alexithymia: what is the relationship?

**DOI:** 10.12688/f1000research.2-81.v2

**Published:** 2013-05-20

**Authors:** Paolo Maria Michetti, Stefano Eleuteri, Marta Giuliani, Roberta Rossi, Chiara Simonelli

**Affiliations:** 1 Department of Urology, Sapienza University of Rome, Roma, 155 – 00161, Italy; 2 Faculty of Medicine and Psychology, Sapienza University of Rome, Roma, 1035 – 00189, Italy; 3 Institute of Clinical Sexology, Roma, 78 - 00198, Italy

**Keywords:** Delayed ejaculation, alexithymia, TAS

## Abstract

Delayed Ejaculation (DE) is probably the least studied and understood of the male sexual dysfunctions (MSD). There is still little unanimity concerning its psychological/interpersonal aetiology. Previous studies found that MSD are strongly related with alexithymia, a multifaceted personality construct that describes a disturbance in the regulation of emotions.The aim of this study was to investigate the presence of alexithymia in men with DE and correlate alexithymia levels with DE severity. According to specific features of the symptoms, we hypothesized that alexithymia would not be correlated with this specific sexual disorder.

54 outpatients with a diagnosis of DE assessed at the Institute of Clinical Sexology and the Urology Department of Sapienza, University in Rome were enrolled in the study. DE was diagnosed after a specialist examination and according to Diagnostic and Statistical Manual of Mental Disorders -IV-TR criteria. Participants were provided with the Toronto Alexithymia Scale (20 items; TAS-20), a self-measure of the Intravaginal Ejaculation Latency Time and an *ad hoc *questionnaire to collect anamnestic data.

9.3% of patients could be categorized as alexithymics, 9.3% of them as borderline, while 81.4% of the sample was found to be non-alexithymic. The overall average TAS-20 score was 45.46. Results show that alexithymia is correlated neither with the presence of DE nor with its severity, in contrast to other MSDs, where this condition was found in about 30% of patients.

The data presented suggest that DE, although not correlated to alexithymia, is probably related to other psychogenic features such as hypercontrol configuration. This paper can contribute to the understanding of DE, by excluding one of the possible etiological factors, previously found to be important in the onset and the maintenance of the other MSDs. More studies are needed in order to better understand DE and provide recommendations about treatment.

## Introduction

Delayed Ejaculation (DE) is probably the least studied and understood of the Male Sexual Dysfunctions (MSD) ^1^. However, its negative impact is significant as it typically results in lack of sexual fulfilment for both the man and his partner; the problem is further increased when procreation is among the couple’s goals of sexual intercourse. The terms delayed, retarded, inadequate or inhibited ejaculation, idiopathic anejaculation (inability to ejaculate) and psychogenic anejaculation have all been used synonymously to describe a delay or absence of male orgasm ^2^. The Diagnostic and Statistical Manual of Mental Disorders -IV-TR ^3^ defines DE as:

“ *the persistent or recurrent delay in, or absence of, orgasm after a normal sexual excitement phase during sexual activity that the clinician, taking into account the person’s age, judges to be adequate in focus, intensity, and duration. The disturbance causes marked distress or interpersonal difficulty*”.

In the literature DE is reported to have an incidence rate between 3% and almost 40%, depending on age and comorbidities ^4– 8^. The essential medical causes of this problem include neurogenic, infective, endocrine and medication factors ^9^. Psychological and interpersonal aspects are also involved in the onset and maintenance of DE, but there is little unanimity concerning these causes ^1^.

Previous studies have found that Hypoactive Sexual Desire Disorder (HSDD), Erectile Dysfunction (ED) and Premature Ejaculation (PE) are strongly correlated with alexithymia ^10– 14^. Alexithymia (from the ancient Greek “a” for lack, “lexis” for word, and “thymos” for emotion) is a multifaceted personality construct introduced in the early 1970s by Nemiah and Sifneos ^15, 16^. The alexithymia construct describes a set of deficits in the cognitive processing of emotions, or more generally, a disturbance in the regulation of emotions ^17^. The personality features that characterize alexithymic individuals include difficulty in identifying emotions and differentiating between emotions and the bodily sensations of emotional arousal; difficulty in communicating emotions to others; reduced imaginal and fantasy activity and an externally oriented cognitive style, a sub-factor of the alexithymia construct involving impoverished fantasy life, utilitarian thinking and a focus on external concrete data of the sensate environment ^18, 19^.

A deep analysis of the literature underlines that:

- 20–25% of subjects presenting MSDs can be classified as alexithymics ^10– 13^;
- there is a significant correlation between alexithymia and the length/severity of MSD symptoms ^12– 14^;
- alexithymia is particularly correlated with HSDD ^10^, ED ^10, 12^ and PE ^13^.

The aim of this study was to investigate the presence of alexithymia in men with DE and to correlate alexithymia levels with DE severity. According to specific features of the symptoms we hypothesize that alexithymia will not be associated with this specific sexual disorder. In fact, emotional control, an important clinical feature identified in DE conceptualization ^20, 21^, has been found to be negatively associated with alexithymia ^22, 23^.

## Methods

60 outpatients with a diagnosis of DE assessed at the Institute of Clinical Sexology and at the Urology Department of Sapienza, University in Rome were enrolled in the study. DE was diagnosed after a specialist examination, i.e. a careful anamnesis and a physical examination, and according to DSM-IV-TR criteria.

Patients with previous and current psychotherapeutic experiences (6 patients) were not included because of the possible influence of psychotherapy on the degree of alexithymia ^19^. Participants were informed in detail about the study, guaranteed confidentiality and anonymity and advised of their right to withdraw at any time. The ones who gave their consent to participate were provided with a research protocol including the following measures. The research protocol received institutional approval.

DE severity was evaluated using a self-reported Intravaginal Ejaculation Latency Time (IELT; ^24^) measure. The participants were given the following options to categorise the latency time: 10–20 mins, 20–30 mins, 30–40 mins, 40–50 mins and ‘never reached orgasm’.

A questionnaire collecting demographic, anamnestic data and characteristics of the symptom (Lifelong or Acquired, Situational or Generalized DE) was filled by all patients. Alexithymia was measured by the 20-item Toronto Alexithymia Scale (TAS-20) ^25, 26^, a validated tool used to evaluate alexithymia levels. Even though alexithymia levels are measured on a continuous scale, different studies have established standard cut-off scores to distinguish alexithymic (TAS-20≥61) from non-alexithymic subjects (TAS-20≤51), with a borderline area for scores between 51 and 61 ^19^. The scale has a factor structure that reflects three separate, yet conceptually related, facets of the alexithymia construct ^25^. The three factors are difficulty identifying feelings and distinguishing them from the somatic sensations that accompany emotional arousal (Subfactor 1), difficulty communicating feelings to other people (Subfactor 2) and externally oriented thinking (Subfactor 3).

Using SPSS version 19 (IBM-SPSS Inc., 2010, Chicago, IL, USA), descriptive analyses were run in order to check sample and symptom characteristics. The relationship between alexithymia level and DE severity was investigated by Spearman’s Rho correlation coefficient.

## Results

Table 1 shows the demographic characteristics of the respondents. The majority were in a relationship, employed, and had attended at least secondary school. Descriptive analysis ( Table 2) revealed that the great majority of the patients presented a lifelong (73.3%) and generalized (90.6%) DE. Moreover, half of the patients declared that they had never reached orgasm, reporting that ejaculation was impossible. 81.4% of the sample was found to be non-alexithymic, with an overall average TAS-20 score of 45.46.

**Table 1.** Demographic characteristics of the respondents.

Description	Value
**Age (Mean ± SD)**	36.31 ± 9.09
**Relationship status (%)**	
Single	20.4
In relationship	79.6
**Professional status (%)**	
Employed	79.2
Unemployed	5.7
Student	15.1
**Education (%)**	
Primary School	9.3
Secondary School	42.6
University	48.1

**Table 2.** Descriptive statistics of patients diagnosed with delayed ejaculation.

Patient characteristic	%
**Delayed ejaculation**	
Lifelong	73.3
Acquired	26.7
Generalized	90.6
Situational	9.4
**IELT**	
10–20 min	9.3
20–30 min	7.4
30–40 min	22.2
40–50 min	11.1
Never reached orgasm	50
**Alexithymia**	
Non-Alexithymic	81.4
Borderline	9.3
Alexithymic	9.3

TAS scores did not significantly correlate with the severity of DE measured by IELT (p = 0.54). IELT -Intravaginal Ejaculation Latency Time.

Updated version 2: Delayed ejaculation and alexithymia: raw data Data comprising demographic characteristics, clinical evaluation of Delayed Ejaculation and TAS-20 scores.

TAS scores did not significantly correlate with the severity of DE measured by IELT (p = 0.54). Total TAS scores and the scores of the three different subfactors did not significantly correlate with the severity of DE measured by IELT (respectively p = 0.54; p = 0.95; p = 0.47; p = 0.24).

## Discussion

Problems with “difficulty” in ejaculating may vary from a delay in the latency to ejaculation to a complete inability to ejaculate (anejaculation). According to the literature, we used the term Delayed Ejaculation (DE) to describe any and all ejaculatory disorders with either a long latency or absence of ejaculation ^3^: our sample was composed of 50% of patients with delayed ejaculation and 50% that could not ejaculate. Furthermore, we found that 73.3% of the sample presented lifelong DE: this data is in strong contrast with the literature, where around 25% of patients suffer from lifelong DE, with the remainder reporting it as acquired ^7, 27, 28^.

According to our hypothesis, the results show that the alexithymia construct does not seem to be correlated with DE (only 9.3% of the patients could be classified as alexithymics), in contrast to other MSDs, where this condition was found in about 30% of patients, specifically it was found in 23% for suffering from HSDD, in 25% of them with PE and 34% of the ones declaring to have ED ^10– 13^.

There is some evidence supporting the inadequacy of alexithymia as an etiological factor in DE pathogenesis. From the first psychodynamic interpretations, DE has been associated with obsessive traits ^20, 21^, in which emotional control has an important role. This hyper-control configuration in men presenting DE has also been strongly confirmed in our psychotherapeutic experience for this disorder. In the clinical setting it is very common that patients with DE present a wide range of control behaviours, which affect not only their sexual experience, but also their daily activities and relations. This control is indiscriminately projected over emotions, behaviour and thoughts. The presence of this control presumes that the person recognizes his emotional responses, a trait that is typically absent in subjects with alexithymia. Indeed alexithymia and emotional control were previously found to be negatively associated ^22, 23^. This finding could explain the low rates of alexithymic traits in our sample.

The lack of difficulty in identifying and communicating emotions to others in patients with DE may also be connected to the greater social acceptance of DE than Premature Ejaculation and Erectile Dysfunction: the symptom does not seem to affect core male identity. From a somatopsychic perspective, the consequent low individual and relational distress arising from DE conditions could represent an important protective factor from creating or exacerbating high levels of alexithymia.

More research is still necessary to understand and treat DE There is a strong need for more studies examining the precipitating and maintaining factors of this problem. Controlled and randomized outcome studies, using validated measures with sufficient follow-up periods, are necessary to determine the psychogenic variables connected with the onset and the maintenance of DE and the most efficacious methods of psychological treatment. The present studies are inadequate to make evidence-based recommendations about treatment ^1^.

This paper can contribute to the understanding of DE by excluding one of the possible etiological factors that has been previously found to be important in the onset and the maintenance of other MSDs.

## Consent

Written informed consent was obtained from all study participants before receiving the research protocol. Each subject was advised of own right to withdraw at any time.
