# Supplementary material for: Delayed ejaculation and alexithymia: what is the relationship?
Source: F1000Res. 2013 May 20;2:81. Originally published 2013 Mar 7. [Version 2] doi: 10.12688/f1000research.2-81.v2 (PMC3917660; doi:10.12688/f1000research.2-81.v2)
Supplement: Peer review history

# Peer review: Referee response for version 2

**DOI:** 10.5256/f1000research.1509.r3991

**Published:** 2014-03-06

**Authors:** Salvatore Caruso

**Affiliations:** 1 Department of Medical Surgical Specialties, University of Catania, Catania, Italy

I have read this submission. I believe that I have an appropriate level of expertise to confirm that it is of an acceptable scientific standard.

# Peer review: Referee response for version 2

**DOI:** 10.5256/f1000research.1509.r960

**Published:** 2013-05-21

**Authors:** Nelson Bennett

**Affiliations:** 1 Institute of Urology, Lahey Clinic Medical Center, Burlington, MA, USA

The manuscript is much improved.

I have read this submission. I believe that I have an appropriate level of expertise to confirm that it is of an acceptable scientific standard.

# Peer review: Referee response for version 1

**DOI:** 10.5256/f1000research.1229.r898

**Published:** 2013-04-17

**Authors:** Salvatore Caruso

**Affiliations:** 1 Department of Medical Surgical Specialties, University of Catania, Catania, Italy

The research is very interesting because it focuses the attention on the relationship between emotional regulation and delayed ejaculation. The emerged results supply an appreciable suggestion for the clinical practice underlining how different male sexual disorders could be influenced by different emotional patterns. 

The self-report questionnaire the Authors used, beyond the total alexithymia score, provides three separate, yet conceptually related, facets of the alexithymia construct: difficulty identifying feelings and distinguishing them from the somatic sensations that accompany emotional arousal (F1), difficulty communicating feelings to other people (F2) and externally oriented thinking (F3).

Despite the data revealed negative correlation between alexithymia and delayed ejaculation, the study would be stronger if the Authors could specify if the same negative trend emerges also correlating delayed ejaculation levels with the three sub-factors

I have read this submission. I believe that I have an appropriate level of expertise to confirm that it is of an acceptable scientific standard.

# Response

**Published:** 2013-05-05

**Authors:** Stefano Eleuteri

**Affiliations:** Dipartimento di Psicologia, University of Rome 'La Sapienza', Italy

Dr. Caruso, these are the results of the Spearman's Rho correlation for the 3 subfactors of TAS 20 , to be included in the 'Results' section: F1 (ρs= - 0.010, p=0.95), F2 (ρs= - 0.101, p=0.47), F3 (ρs= - 0.164, p=0.24).

# Peer review: Referee response for version 1

**DOI:** 10.5256/f1000research.1229.r837

**Published:** 2013-03-14

**Authors:** Nelson Bennett

**Affiliations:** 1 Institute of Urology, Lahey Clinic Medical Center, Burlington, MA, USA

The authors seek to define the relationship of alexithymia to delayed orgasm. This is an excellent research question that needs to be answered in order to advance our knowledge psychosexual disorders. The study design and methods are appropriate. 

The study would be stronger if the results of the Spearman’s Rho correlation were included in the ‘Results' section. This would lend more support to the study conclusions. In reality, without the coefficient data, the data does support not the conclusion of the manuscript.

I have read this submission. I believe that I have an appropriate level of expertise to confirm that it is of an acceptable scientific standard, however I have significant reservations, as outlined above.

# Response

**Published:** 2013-03-20

**Authors:** Stefano Eleuteri

**Affiliations:** Dipartimento di Psicologia, University of Rome 'La Sapienza', Italy

Dr. Bennett, these are the results of the Spearman's Rho correlation, to be included in the 'Results' section: ρs= - 0.086, p=0.54.
